# Diagnostic value of a multimodal approach combining inflammatory biomarkers, tumor markers, and ultrasonographic features for borderline ovarian epithelial tumors

**DOI:** 10.3389/fonc.2026.1758644

**Published:** 2026-03-17

**Authors:** Yaduan Gan, Xiaoling Shen, Yuzhen Wang, Shufen Wu, Ming Chen

**Affiliations:** Department of Ultrasound Medicine, Zhangzhou Affiliated Hospital of Fujian Medical University, Zhangzhou, Fujian, China

**Keywords:** borderline ovarian epithelial tumors (BOETs), CA125, human epididymis protein 4 (HE4), monocyte-to-lymphocyte ratio (MLR), neutrophil-to-lymphocyte ratio (NLR), platelet-to-lymphocyte ratio (PLR), ultrasonography

## Abstract

**Backgrounds:**

The preoperative differentiation of borderline ovarian epithelial tumors (BOETs) remains challenging. While systemic inflammatory markers such as the platelet-to-lymphocyte ratio (PLR), neutrophil-to-lymphocyte ratio (NLR), and monocyte-to-lymphocyte ratio (MLR) have shown prognostic value in ovarian cancer, their diagnostic potential in BOETs and the efficacy of a multimodal panel incorporating them have not been fully elucidated. This study aimed to evaluate the predictive value of combining these inflammatory markers with tumor markers (CA125, HE4) and specific ultrasonographic features for the diagnosis of BOETs.

**Results:**

The study enrolled 301 patients (101 benign, 99 BOETs, 101 malignant). The NLR and MLR in the BOETs group were significantly higher than in the benign group but lower than in the malignant group (*P* < 0.001). CA125 and HE4 levels in the BOETs group were higher than in the benign group but substantially lower than in the malignant group (*P* < 0.05). The microcystic pattern was a highly specific feature for BOETs, with its prevalence being significantly higher in the borderline group (53.54%) compared to the benign (0.99%) and malignant (6.93%) groups (*P* < 0.001). The prevalence of a predominantly solid component, rich blood supply, irregular tumor morphology, and ascites showed a progressive increase from the benign to the borderline and malignant groups, with significant differences among all three (*P* < 0.001). The combination of inflammatory and tumor markers yielded AUCs of 0.714 for differentiating benign from borderline tumors and 0.861 for malignant from borderline tumors, outperforming individual markers (*P* < 0.05). Combining the microcystic pattern with the count of other positive sonographic features significantly improved diagnostic performance over either feature alone for both benign vs. borderline (AUC = 0.886) and malignant vs. borderline (AUC = 0.914) differentiations (*P* < 0.05). The comprehensive multimodal approach (inflammatory markers, tumor markers, microcystic pattern, and count of positive sonographic features) demonstrated robust diagnostic efficacy, with an AUC of 0.914 for benign vs. borderline differentiation and an AUC of 0.933 for malignant vs. borderline differentiation (*P* < 0.001).

**Conclusions:**

A multimodal approach integrating PLR/NLR/MLR, CA125, HE4, and the ultrasonographic microcystic pattern significantly enhances the preoperative differentiation of BOETs, and reveal that BOETs have biological characteristics of “inflammatory activation” to a certain extent.

## 1 Introduction

Borderline ovarian epithelial tumors (BOETs) are a distinct category of neoplasms with low malignant potential, exhibiting biological behavior intermediate between benign and malignant tumors. They account for approximately 4% to 14% of all ovarian epithelial neoplasms (1). Compared to invasive ovarian cancer, BOETs generally have a favorable prognosis, with a 5-year survival rate of 95%; however, some cases may recur or progress to low-grade serous carcinoma (2), mucinous carcinoma (3), or low-grade endometrioid carcinoma (1). The accurate preoperative differentiation of BOETs remains a clinical challenge, as their clinical and imaging features often overlap with those of benign ovarian tumors or early-stage ovarian cancer (4). Currently, the preoperative assessment of ovarian masses primarily relies on ultrasound examination, tumor markers (e.g., CA125, HE4), magnetic resonance imaging, and clinical experience. However, the limited sensitivity and specificity of any single modality can lead to misdiagnosis or unnecessary surgical interventions (5). BOT management differs from both benign lesions and invasive carcinoma, particularly regarding fertility-sparing decisions (the target population is younger women of reproductive age) and the extent of staging surgery (6, 7). Therefore, exploring more reliable combined diagnostic approach is crucial for optimizing the clinical management of BOETs.

In terms of imaging, ultrasound is a non-invasive, cost-effective, and reproducible modality, making it an excellent tool for differentiating between benign and malignant ovarian masses. The ADNEX model, developed by the International Ovarian Tumor Analysis (IOTA) group, has demonstrated strong performance in distinguishing benign from malignant tumors, yet its utility in diagnosing BOETs has certain limitations (8, 9). CA125 is the most widely used tumor marker for ovarian neoplasms, but its levels in BOETs may be lower than in malignant ovarian cancer and can be confounded by benign conditions such as endometriosis and pelvic inflammatory disease (10, 11). Human epididymis protein 4 (HE4), an emerging biomarker, has shown diagnostic efficacy comparable to CA125 for ovarian cancer (12), but its predictive value in the diagnosis of BOETs is limited (13).

In recent years, the role of systemic inflammatory response in tumorigenesis and progression has garnered significant attention. The platelet-to-lymphocyte ratio (PLR), neutrophil-to-lymphocyte ratio (NLR), and monocyte-to-lymphocyte ratio (MLR), as indicators of the body’s inflammatory state, have been shown to correlate with tumor progression and prognosis in various malignancies, including gastrointestinal, lung, and head and neck cancers (14–16). Research indicates that inflammatory cells within the tumor microenvironment can promote invasion and metastasis by facilitating angiogenesis and suppressing immune responses (17).Several studies have already confirmed the significant value of PLR, NLR, and MLR in the clinical diagnosis and prognostic prediction of ovarian cancer (18–22). However, the diagnostic utility of these inflammatory markers specifically for BOETs has not been thoroughly investigated, and their potential when combined with established tumor markers (e.g., CA125, HE4) and ultrasonographic features remains to be explored.

This study aims to evaluate the predictive value of PLR, NLR, and MLR in combination with CA125, HE4, and ultrasonographic features for the diagnosis of BOETs, with the goal of providing a more reliable preoperative risk assessment tool. Through a retrospective analysis of patient data, we aimed to evaluate the diagnostic efficacy of a multiparametric multimodal panel and compare its performance with that of individual single indicators. This research may not only help optimize individualized diagnosis and treatment strategies for BOETs, but also provides clinical evidence for understanding the systemic inflammatory response in ovarian tumors of low malignant potential.

## 2 Materials and methods

### 2.1 Patient and data collection

This retrospective study was approved by the Ethics Review Committee of Zhangzhou Affiliated Hospital of Fujian Medical University (2025LWB283), and the requirement for written informed consent was waived. The study population consisted of patients diagnosed with ovarian tumors confirmed by surgical or biopsy pathology at our hospital between January 2019 and January 2025. The primary inclusion criterion was the presence of an adnexal mass identified on ultrasound with complete data. If multiple lesions were present in the adnexal area, the one with the most complex sonographic appearance was selected; if the appearances were similar, the largest lesion was chosen. Exclusion criteria were as follows: (1) patients who did not undergo an ultrasound examination at our hospital within one month prior to surgery; (2) patients with unclear ultrasound images or incomplete reports from which the required features could not be extracted; (3) patients with a previous history of radiotherapy or chemotherapy; (4) patients with incomplete clinical or serological data (e.g., blood counts, CA125, HE4); (5) patients with unclear postoperative pathology reports; (6) patients whose pathology revealed two or more histological types within the same lesion; (7) concurrent pregnancy; and (8) patients with complicated acute infections or diseases involving systemic inflammatory markers, including chronic inflammatory diseases (such as rheumatoid arthritis, inflammatory bowel disease), autoimmune diseases, chronic liver diseases, and those using steroids or other immunosuppressive drugs. Clinical, ultrasonographic, and pathological data were extracted from inpatient or outpatient records. A total of 301 patients were ultimately included: 99 with BOETs, 101 with malignant tumors, and 101 with benign tumors collected randomly from the same period.

### 2.2 Ultrasound data acquisition

**Figure 1:**
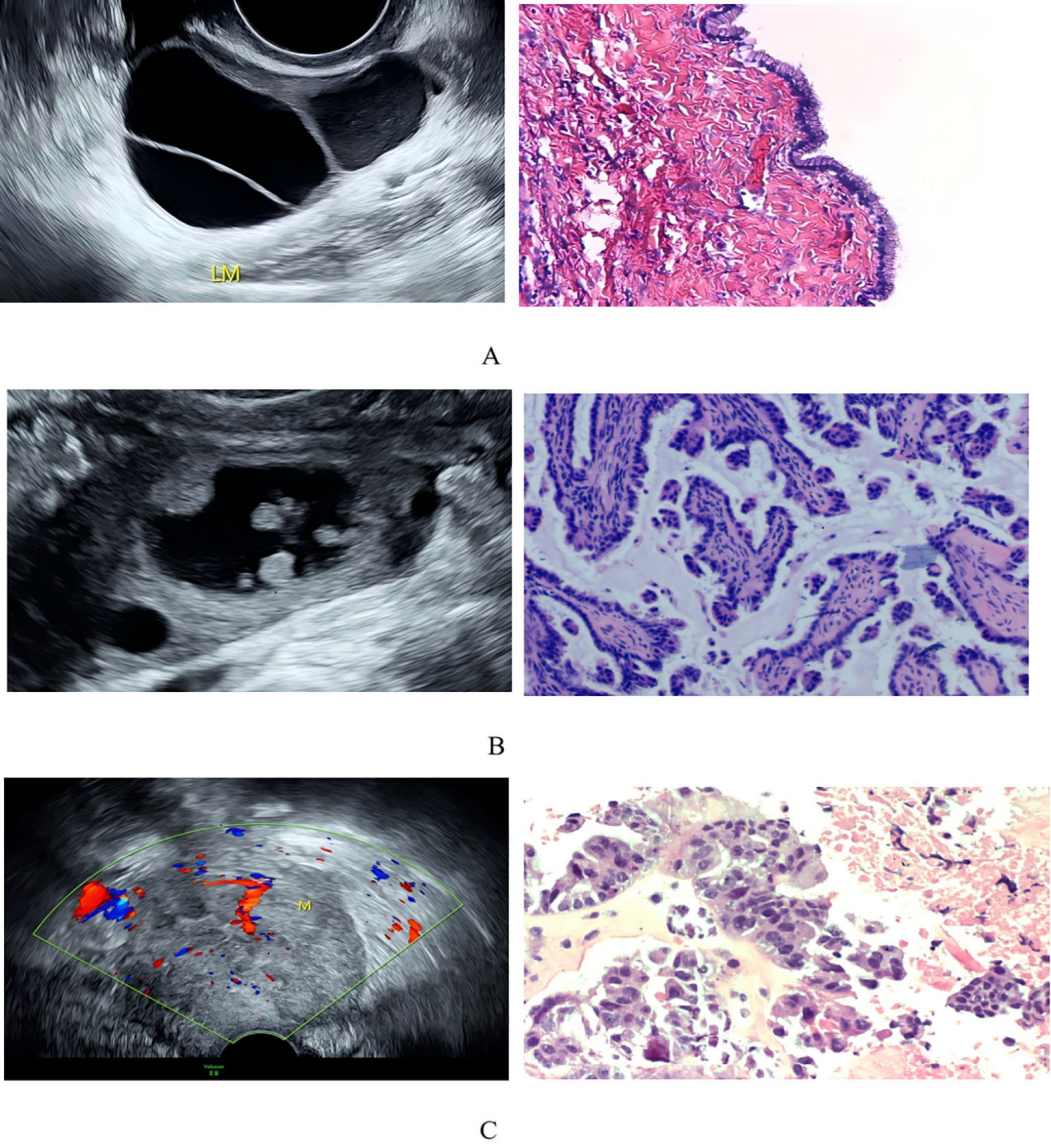
Representative ultrasound and histopathology of ovarian tumors. **(A)** Benign mucinous cystadenoma: Ultrasound shows a multilocular cyst with thin septa. Histology (H&E, ×100) shows tall columnar cells with apical mucin. **(B)** Serous borderline tumor: Ultrasound shows a cyst with papillary projections and microcystic pattern (MCP). Histology (H&E, ×100) shows branching papillae with mild nuclear atypia. **(C)** High-grade serous carcinoma (HGSC): Ultrasound shows a solid mass with rich blood supply. Histology (H&E, ×100) shows necrosis and atypical epithelial cell nests.

Ultrasound examinations were performed using color Doppler ultrasound systems, including the GE Voluson E8 and Mindray Resona I9, equipped with a 3.5-6.5 MHz abdominal probe and a 5–9 MHz transvaginal probe. Ultrasound images or dynamic videos from eligible patients were retrieved, and sonographic features were recorded. Two physicians, each with over five years of experience in gynecological ultrasound diagnosis, independently reviewed the images or videos in a blinded manner. They documented features according to the International Ovarian Tumor Analysis (IOTA) terminology (23) including tumor location, size, morphology, septal thickness, number of locules, number of papillary projections, proportion of solid components, blood supply, and the presence of ascites, with special attention to the characteristic microcystic pattern (MCP). Any disagreements between the two reviewers were resolved through discussion to reach a consensus. The final results were further confirmed by an expert with over 10 years of experience in gynecological ultrasound. The gold standard for diagnosis was the postoperative histopathological result. The MCP was defined as a thin-walled cystic structure, 1–3 mm in size, located within the solid component, papillary projections, or on the cyst wall or septum (24). A papillary projection is any solid protrusion into the cyst cavity from the cyst wall or a solid element on a septum with a height of ≥3 mm (23). Papillary projections with a height of ≥7 mm were defined as large, derived from the IOTA Simple Rules where a solid component <7 mm indicates a benign feature (25). A septum with a thickness of ≥3 mm was defined as thick, while <3 mm was thin (23). A lesion was considered predominantly solid if ≥50% of it consisted of solid components. The blood flow score was defined as follows: 1 for no flow, 2 for minimal flow, 3 for moderate flow, and 4 for abundant flow. For classification and statistical analysis, scores of 1 and 2 were categorized as “scant blood flow,” while scores of 3 and 4 were categorized as “rich blood flow.” “Irregular” morphology was defined by the presence of any of the following: papillary projections, solid components, or a non-smooth cyst wall. Ascites was defined as fluid detected outside the pouch of Douglas. **Figure 1** demonstrates representative ultrasonographic features and corresponding histopathological images of benign, borderline, and malignant ovarian tumors, respectively.

### 2.3 Statistical methods

Statistical analyses were performed using SPSS version 25.0, and figures were generated with R software version 4.0. Categorical data were described as counts (percentages) and compared among the three groups using the chi-square test or Fisher’s exact test. Continuous data, which did not conform to normal distribution and homogeneity of variance, were presented as medians [interquartile range] and compared using the Kruskal-Wallis H test followed by Bonferroni-corrected multiple comparisons. Categorical data were compared between the two groups using the Chi-square test or Fisher’s exact test, while continuous data were analyzed using the Student’s t-test or the Mann-Whitney U test. Receiver operating characteristic (ROC) curves were constructed, and the area under the ROC curve (AUC) was calculated to evaluate the predictive value of the parameters. The cut-off value was determined by maximizing the Youden’s index of the ROC curve. Differences between AUC values were assessed using the Delong test, with all multiple comparisons in this test performed via Bonferroni correction. A *P*-value <0.05 was considered statistically significant.

## 3 Results

A total of 301 patients with ovarian epithelial tumors were included in this study, comprising a benign group (n=101), a borderline group (n=99), and a malignant group (n=101). There was a significant difference in age distribution among the three groups *(P* < 0.001): the median age was 36 years (IQR: 31-46) in the benign group, 43 years (IQR: 30.5-56) in the borderline group, and 54 years (IQR: 48-61) in the malignant group. The proportion of postmenopausal patients was significantly higher in the malignant group (66.3%) compared to the borderline (37.4%) and benign groups (11.9%) (*P* < 0.001) (**Table 1**).

**Table 1:** Clinical characteristics of patients with different pathological types of tumors.

Characteristics	Benign(N=101)	Borderline (N=99)	Malignant (N=101)	*P*
Age (years)	36.00 [31.00;46.00]	43.00 [30.50;56.00]*	54.00 [48.00;61.00]*#	<0.001
Pre/postmenopausal (cases)	88/12	62/37	37/67	<0.001
Personal history of cancer	97/4	97/2	93/8	0.154

* *P* < 0.05 compared with benign tumors.

# *P* < 0.05 compared with BOETs.

**Figure 2:**
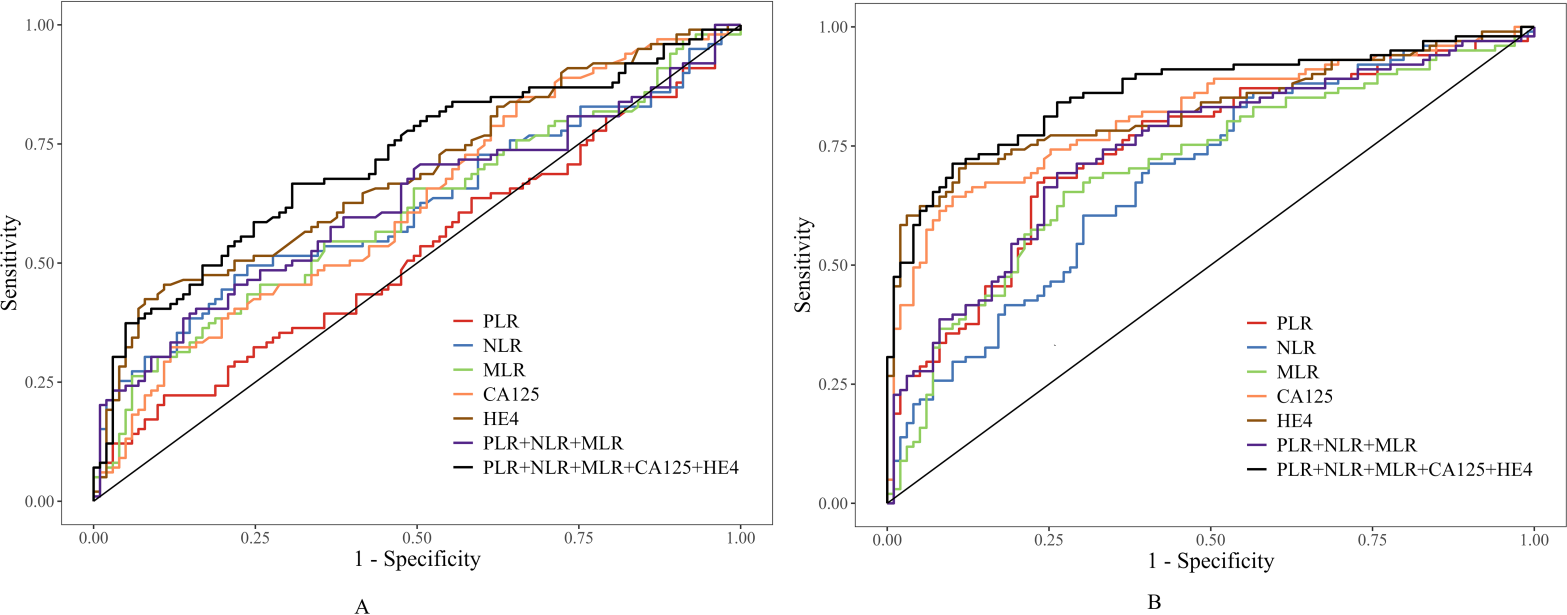
Two side-by-side receiver operating characteristic (ROC) curve graphs compare diagnostic performance for multiple biomarkers: PLR, NLR, MLR, CA125, HE4, their three-way combination, and the combination of all five. Both panels plot sensitivity versus one minus specificity, with each line representing a different biomarker or combination, as indicated in the colored legend. Panel A and Panel B represent different test conditions or groups. All curves show varying performance, with the black line, representing the five-marker combination, achieving the highest sensitivity across most specificity ranges.

As shown in **Table 2**, significant differences were observed in all serological markers among the three groups (all *P* < 0.05). Specifically, PLR, NLR, and MLR in the malignant group were all significantly higher than in the borderline and benign groups (all *P* < 0.05). The NLR and MLR in the borderline group were significantly higher than in the benign group (*P* < 0.05), but there was no statistically significant difference in PLR between these two groups (*P*>0.05). CA125 and HE4 levels were extremely high in the malignant group (medians were 27.6 times and 7.7 times that of the benign group, respectively). The median CA125 and HE4 levels in the borderline group were significantly higher than in the benign group *(P* < 0.05) but substantially lower than in the malignant group (*P* < 0.05).The diagnostic efficacy of serological markers for benign, borderline and malignant ovarian epithelial tumors was evaluated by ROC curve analysis, as shown in **Figure 2**.

**Table 2:** Serological markers of patients with different pathological types of tumors.

Serological markers	Benign(N=101)	Borderline(N=99)	Malignant (N=101)	*P*
RBC(10^9^/L)	4.55 [4.34;4.83]	4.41 [4.07;4.62]*	4.32 [3.99;4.64]*	<0.001
WBC(10^9^/L)	6.50 [5.43;7.38]	6.78 [5.70;8.20]	7.64 [6.47;9.57]*#	<0.001
HGB(g/L)	131.00 [123.00;140.00]	126.00 [117.00;134.50]	123.00 [112.00;135.00]*	0.003
NEU(10^9^/L)	3.84 [3.32;4.70]	4.32 [3.37;5.52]*	5.48 [4.28;7.31]*#	<0.001
LYM(10^9^/L)	1.87 [1.56;2.19]	1.74 [1.46;2.13]	1.46 [1.13;1.92]*#	<0.001
MO(10^9^/L)	0.31 [0.25;0.39]	0.36 [0.26;0.41]	0.46 [0.34;0.60]*#	<0.001
PLT(10^9^/L)	275.00 [231.00;314.00]	261.00 [219.00;316.00]	336.00 [275.00;428.00]*#	<0.001
PLR	147.18 [118.52;177.95]	148.08 [115.40;189.84]	223.23 [167.35;339.71]*#	<0.001
NLR	1.99 [1.67;2.51]	2.46 [1.75;3.92]*	3.56 [2.55;6.18]*#	<0.001
MLR	0.17 [0.13;0.21]	0.20 [0.15;0.27]*	0.30 [0.20;0.45]*#	<0.001
CA125 (U/mL)	21.40 [10.00;40.40]	27.10 [14.35;75.55]*	590.90 [71.30;1638.98]*#	<0.001
HE4 (pmol/L)	36.00 [29.10;42.80]	44.20 [33.50;59.65]*	278.00 [60.60;706.80]*#	<0.001

*RBC* red blood cell count, *WBC* white blood cell count, *HGB* Hemoglobin, *NEU* absolute neutrophil count, *LYM* absolute lymphocyte count, *MO* absolute monocyte count, *PLT* blood platelet count, *PLR* platelet to lymphocyte ratio, *NLR* neutrophil to lymphocyte ratio, *MLR* monocyte to lymphocyte ratio, *CA125* cancer antigen 125, *HE4* human epididymis protein 4.

* *P* < 0.05 compared with benign tumors.

# *P* < 0.05 compared with BOETs.

**Figure 3:**
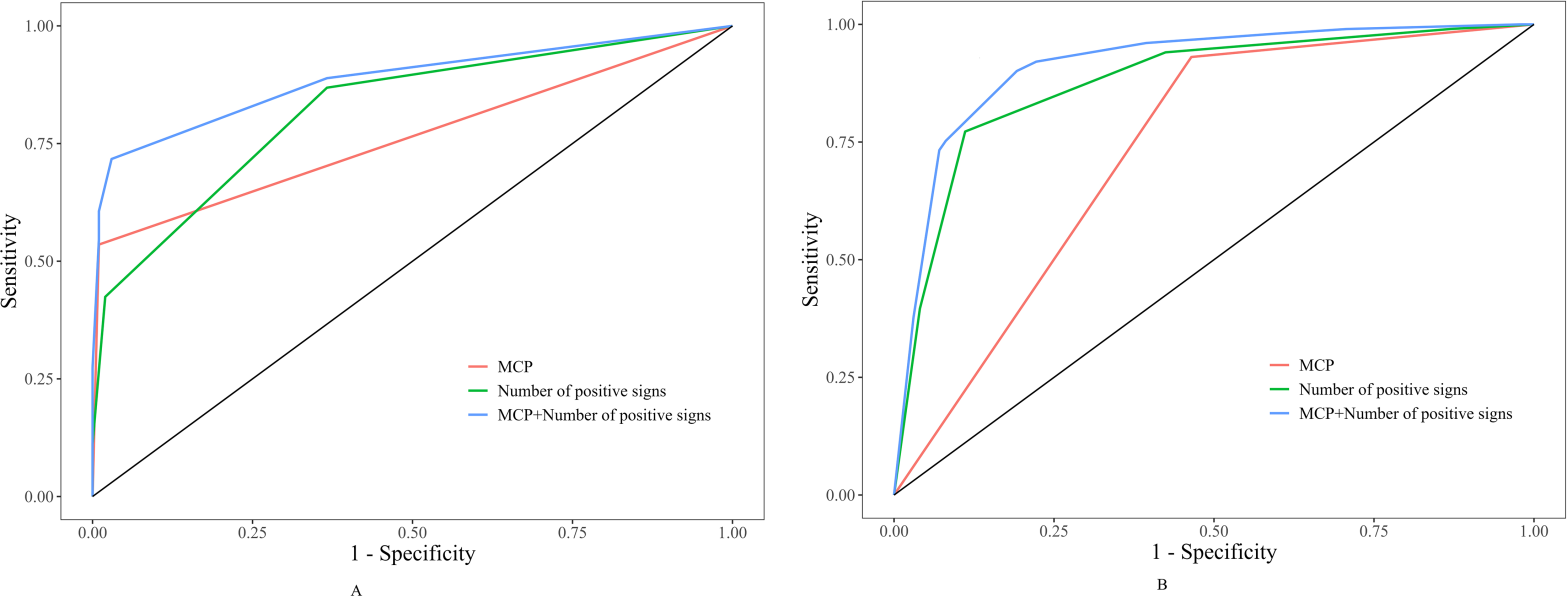
Two side-by-side ROC curve charts labeled A and B display sensitivity versus one minus specificity for three diagnostic approaches: MCP (red), Number of positive signs (green), and MCP plus Number of positive signs (blue). Blue lines demonstrate the highest performance in both plots.

Analysis of ultrasonographic features across different pathological types (**Table 3**) revealed significant differences among the three groups for all features except for the presence of large papillary projections (all *P* < 0.001). Notably, the microcystic pattern was a specific marker for borderline tumors, with its prevalence in the borderline group (53.54%) being significantly higher than in the benign (0.99%) and malignant (6.93%) groups (*P* < 0.001). For features such as predominantly solid composition, rich blood supply, irregular morphology, and ascites, there was a progressive, stepwise increase in prevalence from the benign to the borderline and malignant groups, with statistically significant differences among all three (*P* < 0.001). The presence of thick septa was significantly more common in the borderline and malignant groups than in the benign group, with no significant difference between the borderline and malignant groups. Papillary projections were significantly more frequent in the borderline group compared to both the benign and malignant groups (*P* < 0.05), while the presence of large papillary projections showed no intergroup difference. The diagnostic efficacy of sonographic features for benign, borderline and malignant ovarian epithelial tumors was evaluated by ROC curve analysis, as shown in **Figure 3**.

**Table 3:** Ultrasonographic features of patients with different pathological types of tumors.

Ultrasonographic features	Benign (N=101)	Borderline(N=99)	Malignant (N=101)	*P*
Tumor Size	58.00 [45.00;73.00]	127.00 [73.50;181.00]*	100.00 [72.00;138.00]*	<0.001
Present MCP	1 (0.99%)	53 (53.54%)*	7 (6.93%)#	<0.001
Predominantly solid composition	1 (0.99%)	15 (15.15%) *	74 (73.27%) *#	<0.001
Rich blood supply	1 (0.99%)	36 (36.36%)*	85 (84.16%) *#	<0.001
Irregular morphology	37 (36.63%)	86 (86.87%) *	97 (96.04%) *#	<0.001
Ascites	0 (0.00%)	6 (6.06%)*	57 (56.44%)*#	<0.001
Present septa	34 (34.00%)	60 (61.22%) *	37 (49.33%)	0.001
Thick septa	6 (17.65%)	49 (81.67%)*	29 (78.38%) *	<0.001
Present papillary projections	9 (9.00%)	64 (65.31%) *	18 (24.00%) *#	<0.001
Present large papillary projections	9 (90.00%)	53 (82.81%)	17 (94.44%)	0.577

*MCP* microcystic pattern.

* *P* < 0.05 compared with benign tumors.

# *P* < 0.05 compared with BOETs.

Regarding the efficacy of serological markers (including inflammatory and tumor markers) for diagnosing BOETs (**Table 4**), HE4 alone demonstrated the best performance in differentiating benign from borderline tumors (AUC = 0.686, sensitivity 45.5%, specificity 89.1%). This was significantly better than PLR alone (*P* < 0.05), but not statistically different from CA125, NLR, or MLR alone. The combination of PLR/NLR/MLR with CA125/HE4 increased the AUC to 0.714, which was significantly higher than any individual serological marker. In differentiating malignant from borderline tumors, the diagnostic performance ranked from highest to lowest was HE4, CA125, and the PLR/NLR/MLR combination (AUC = 0.826, 0.814, and 0.743, respectively; *P* < 0.05). Combining PLR/NLR/MLR with CA125/HE4 elevated the AUC to 0.861 (sensitivity 71.3%, specificity 89.9%), significantly outperforming the inflammatory markers or tumor markers alone (*P* < 0.05).

**Table 4:** Diagnostic efficacy of serological markers for BOETs.

Comparison & Variables		AUC	95%CI of AUC		Cutoff	*P*	ACC	SEN	SPE
Benign vs. borderline	PLR	0.522	0.441	0.603	214.01	0.705	0.56	0.222	0.891
	NLR^a^	0.615	0.536	0.694	2.54	0.003	0.63	0.495	0.762
	MLR	0.605	0.526	0.684	0.24	0.005	0.605	0.303	0.901
	CA125^a^	0.621	0.543	0.698	64.90	0.002	0.605	0.323	0.881
	HE4^a^	0.686	0.613	0.760	46.70	<0.001	0.675	0.455	0.891
	PLR+NLR+MLR	0.625	0.546	0.703	0.53	0.001	0.625	0.404	0.842
	PLR+NLR+MLR+CA125+HE4^abcdf^	0.714	0.642	0.786	0.46	<0.001	0.68	0.667	0.693
Malignant vs.borderline	PLR	0.743	0.674	0.812	193.83	<0.001	0.72	0.673	0.768
	NLR	0.687	0.614	0.760	2.75	<0.001	0.655	0.713	0.596
	MLR	0.707	0.635	0.780	0.24	<0.001	0.69	0.653	0.727
	CA125^bc^	0.814	0.754	0.875	223.95	<0.001	0.77	0.644	0.899
	HE4^abc^	0.826	0.767	0.886	82.35	<0.001	0.795	0.703	0.889
	PLR+NLR+MLR	0.743	0.674	0.812	0.47	<0.001	0.715	0.693	0.737
	PLR+NLR+MLR+CA125+HE4^abcdf^	0.861	0.809	0.914	0.42	<0.001	0.805	0.713	0.899

Assessment of AUC Differences: Superscript letters denote statistically significant differences *(P* < 0.05): ^a^ vs. PLR; ^b^ vs. NLR; ^c^ vs. MLR; ^d^ vs. CA125; ^f^ vs. the combination of PLR + NLR + MLR.

**Figure 4:**
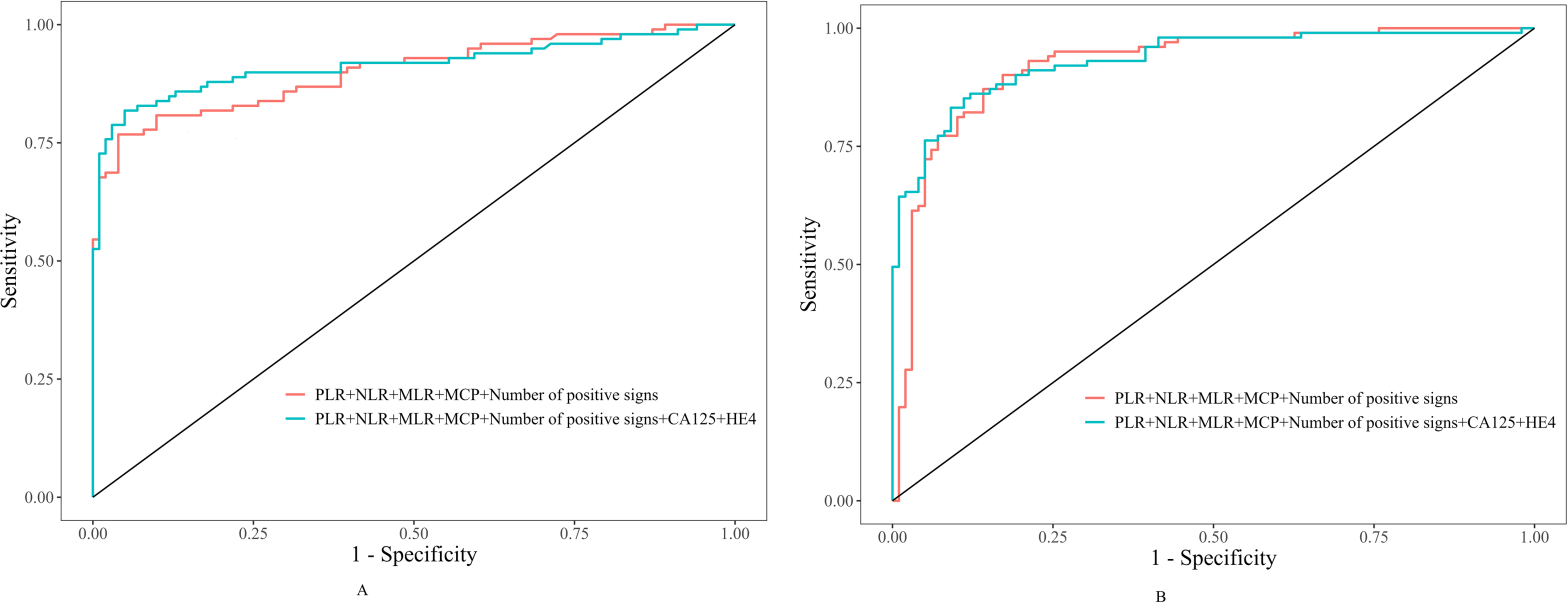
Two side-by-side ROC curve charts labeled A and B compare diagnostic performance. Both charts plot sensitivity versus one minus specificity. Each chart features two curves: one for the combination of PLR, NLR, MLR, MCP, and number of positive signs, and a second for the same combination plus CA125 and HE4, with the latter curve showing slightly improved sensitivity and specificity in both cases.

The “number of positive signs” was defined as the count of positive findings among five features: MCP, predominantly solid, rich blood supply, irregular morphology, and ascites. As shown in **Table 5**, the MCP alone had high specificity but low sensitivity for differentiating benign, borderline, and malignant tumors. Combining the MCP with the number of positive signs markedly improved the diagnostic performance over either the MCP or the number of positive signs alone for both benign vs. borderline (AUC = 0.886) and malignant vs. borderline (AUC = 0.914) differentiations (*P* < 0.05). **Table 6** shows that the multiparametric panel combining serological markers (PLR/NLR/MLR + CA125/HE4) with ultrasonographic features (MCP + number of positive signs) achieved excellent diagnostic performance. For benign/borderline differentiation, the AUC reached 0.914 (sensitivity 81.8%, specificity 95.0%), and for malignant/borderline differentiation, the AUC was 0.933 (sensitivity 83.2%, specificity 90.9%) (*P* < 0.001).The diagnostic performance of the combination of serological markers and sonographic features in the diagnosis of benign, borderline and malignant ovarian epithelial tumors was evaluated by ROC curve analysis, as shown in **Figure 4**.

**Table 5:** Diagnostic efficacy of sonographic features for BOETs.

Comparison & Variables		AUC	95%CI of AUC		Cutoff	*P*	ACC	SEN	SPE
Benign vs. borderline	Present MCP	0.763	0.712	0.813	/	<0.001	0.765	0.535	0.99
	Number of positive signs	0.821	0.769	0.874	0.5	<0.001	0.75	0.869	0.634
	Present MCP+number of positive signs^ab^	0.886	0.84	0.932	0.576	<0.001	0.845	0.717	0.97
Malignant vs.borderline	Present MCP	0.733	0.678	0.788	/	<0.001	0.735	0.535	0.931
	Number of positive signs^a^	0.877	0.829	0.925	2.5	<0.001	0.83	0.772	0.889
	Present MCP+number of positive signs^ab^	0.914	0.873	0.954	0.457	<0.001	0.855	0.901	0.808

The “number of positive signs” was defined as the count of positive findings among five features: MCP, predominantly solid, rich blood supply, irregular morphology, and ascites. Assessment of AUC Differences: Superscript letters denote statistically significant differences (P < 0.05): ^a^ vs. present MCP; ^b^ vs. number of positive signs.

**Table 6:** Diagnostic efficacy of combined serological markers and sonographic features for BOETs.

Comparison & Variables		AUC	95%CI of AUC		Cutoff	*P*	ACC	SEN	SPE
Benign vs. borderline	PLR+NLR+MLR+MCP+number of positive signs	0.886	0.840	0.932	0.58	<0.001	0.845	0.717	0.970
	PLR+NLR+MLR+MCP+number of positive signs+CA125+HE4	0.914	0.870	0.958	0.50	<0.001	0.885	0.818	0.950
Malignant vs.borderline	PLR+NLR+MLR+MCP+number of positive signs	0.914	0.873	0.954	0.46	<0.001	0.855	0.901	0.808
	PLR+NLR+MLR+MCP+number of positive signs+CA125+HE4	0.933	0.899	0.967	0.50	<0.001	0.87	0.832	0.909

The “number of positive signs” was defined as the count of positive findings among five features: MCP, predominantly solid, rich blood supply, irregular morphology, and ascites.

Among the 99 cases of BOETs included in this study, 35 (35.4%) were serous, 54 (54.5%) were mucinous, and 10 (10.1%) were of other pathological types. Due to the limited number of cases with other pathological subtypes, the analysis focused on comparing the two main subtypes of BOETs (serous and mucinous). As shown in **Table 7**, no statistically significant differences were observed between the two groups in inflammatory markers (PLR, NLR, MLR) or HE4 levels. The proportion of postmenopausal patients was significantly higher in the mucinous group (*P* < 0.05). CA125 levels were significantly elevated in the serous group compared to the mucinous group (median 43.90 vs. 24.65, *P* < 0.05). Tumor size was significantly larger in the mucinous group (median 154.50 vs. 77.00), and predominantly solid composition was more frequent in the mucinous group *(P* < 0.05). No significant differences were found in the microcystic pattern, rich blood supply, irregular morphology, or ascites between the two groups.

**Table 7:** Comparison of BOETs by different pathological subtypes (serous and mucinous).

Variables	Pathological subtypes		*P*
	Serous	Mucinous	
	35.4%(35/99)	54.5%(54/99)	/
Age (years)	42.26 ± 13.98	45.65 ± 18.79	0.363
Postmenopausal	8 (22.86%)	25 (46.30%)	0.025
RBC(10^9^/L)	4.44 [4.20;4.66]	4.36 [3.85;4.61]	0.111
WBC(10^9^/L)	6.93 [6.12;8.57]	6.70 [5.56;8.08]	0.209
HGB(g/L)	128.00 [123.00;132.00]	124.50 [113.25;135.00]	0.364
NEU(10^9^/L)	4.60 [3.65;5.68]	4.22 [3.26;5.22]	0.392
LYM(10^9^/L)	1.77 [1.37;2.12]	1.73 [1.37;2.14]	0.678
MO(10^9^/L)	0.33 [0.25;0.37]	0.38 [0.26;0.44]	0.161
PLT(10^9^/L)	271.00 [219.00;322.00]	261.00 [215.25;323.75]	0.775
PLR	141.11 [111.70;191.68]	149.87 [121.40;210.34]	0.491
NLR	2.28 [1.72;4.00]	2.57 [1.76;3.82]	0.903
MLR	0.18 [0.14;0.26]	0.20 [0.15;0.30]	0.328
CA125(U/mL)	43.90 [16.85;232.25]	24.65 [14.00;66.47]	0.035
HE4(pmol/L)	41.80 [33.70;53.95]	50.15 [35.10;60.50]	0.255
Tumor size	77.00 [68.00;128.50]	154.50 [113.00;214.50]	<0.001
Present MCP	18 (51.43%)	31 (57.41%)	0.580
Predominantly solid composition	1 (2.86%)	12 (22.22%)	0.012
Rich blood supply	10 (28.57%)	21 (38.89%)	0.318
Irregular morphology	27 (77.14%)	50 (92.59%)	0.055
Ascites	2 (5.71%)	4 (7.41%)	1.000

## 4 Discussion

This study integrated inflammatory markers (PLR/NLR/MLR), tumor markers (CA125/HE4), and specific ultrasound features (notably, the MCP) into a multimodal afor the preoperative differentiation of BOETs. Our results demonstrate that this combined approach significantly enhances diagnostic performance, surpassing existing single-modality methods (all *P* < 0.05). The findings are discussed below from four perspectives.

### 4.1 The inflammatory microenvironment and biomarker profile of BOETs

This study reveals that BOETs have biological characteristics of “inflammatory activation” to a certain extent. NLR and MLR levels were significantly higher than in the benign group but lower than in the malignant group (*P* < 0.001). This may be linked to the degree of recruitment of tumor-associated neutrophils (TANs) and tumor-associated macrophages (TAMs). As key architects of the pro-tumoral microenvironment, TAMs and TANs drive tumor progression through mechanisms like angiogenesis, immune suppression, and metastasis promotion (26). The inflammatory response in BOETs may be more confined to the local microenvironment, manifesting as a modest elevation in peripheral blood NLR/MLR. Our findings show that the PLR in the BOETs group was not different from the benign group but was significantly lower than in the malignant group. Stone et al. demonstrated that in patients with invasive ovarian carcinoma, IL-6-mediated thrombocytosis promotes an inflammatory microenvironment and tumor progression (27). This aligns with the elevated PLR observed in our malignant group. The insignificant difference in PLR between benign and borderline tumors may reflect their shared biological features. Previous studies have demonstrated that PLR elevation is associated with systemic inflammatory responses in the tumor microenvironment (28, 29). Specifically, Polat et al. found that neutrophil-to-lymphocyte and platelet-to-lymphocyte ratios increase in ovarian tumors only in the presence of frank stromal invasion, and these ratios are weak predictors for borderline cases without invasive characteristics (29). This observation is consistent with our findings, as borderline ovarian tumors characteristically lack stromal invasion, which may explain the similar PLR levels between benign and borderline groups in our cohort. In differentiating benign, borderline, and malignant tumors, nearly all serological markers showed statistically significant differences (except for PLR in benign vs. borderline) and exhibited an increasing trend. This is consistent with the findings of Huang et al. (28) whose study indicated a steady increase in PLR and NLR across benign, borderline, and malignant ovarian epithelial tumors. The characteristics of the inflammatory microenvironment of BOETs remain to be further verified by immunohistochemical staining for detecting the infiltration of immune cells such as CD3+ and CD8+ in tumor tissues.

CA125 and HE4 levels were extremely high in the malignant group and were also elevated in the borderline group compared to the benign group (*P* < 0.05) (**Table 2**). Among the serological markers, HE4 alone showed the best diagnostic performance, superior to individual inflammatory markers, but its efficacy was not statistically different from CA125 alone (**Table 4**). A study by Samborski et al. (12) involving 129 patients confirmed that HE4 has comparable efficacy to CA125 in the workup of ovarian epithelial tumors, which is consistent with our results. In our study, the sensitivity of HE4 for diagnosing BOETs was low, particularly in differentiating the benign and borderline groups, which is consistent with previous studies (30, 31). The relatively lower sensitivity of HE4 in borderline tumors may be explained by several factors. In invasive ovarian cancers, HE4 expression has been associated with higher proliferative and invasive potential (32).Borderline tumors, however, are characterized by limited malignant potential and absence of stromal invasion (33).Furthermore,HE4 expression in ovarian tumors is heterogeneous and influenced by multiple factors including histological subtype and menopausal status (34, 35). These variables, which were not fully stratified in our analysis, may affect HE4 secretion and thus impact its diagnostic sensitivity in the borderline cohort. Therefore, while the lower proliferative and invasive nature of borderline tumors might be one contextual factor, the interpretation of HE4 levels in this group should also consider histopathological heterogeneity and relevant clinical confounders. Further research is needed to clarify the regulatory mechanisms of HE4 expression specifically in borderline ovarian tumors.

Further comparison between the two main pathological subtypes of BOETs (serous and mucinous) revealed no significant differences in multiple inflammatory markers (e.g., PLR, NLR, MLR), suggesting that while inflammatory indicators may hold value in assessing the malignant potential of ovarian tumors, they offer limited utility in distinguishing between different pathological subtypes of BOETs. On the other hand, CA125 levels were significantly higher in the serous group than in the mucinous group, whereas no statistically significant difference was observed in HE4 levels between the two groups. These findings indicate that current biomarkers have limited effectiveness in differentiating pathological subtypes, especially in identifying other rare subtypes, it will be necessary to explore more specific novel indicators and carry out multicenter studies to achieve accurate differentiation of different pathological types of ovarian tumors.

### 4.2 Diagnostic value of ultrasonographic features

The prevalence of the MCP was significantly higher in BOETs than in benign and malignant tumors (**Table 3**), a finding likely rooted in its pathology. The MCP arises from cystic degeneration at the tips of papillary structures, forming small fluid-filled cavities of 1–3 mm (24). Liu et al. (36) found that the proportion of MCP was higher in serous borderline tumors than in other pathological types. Their analysis of 55 pathological images revealed that 21 cases exhibited a “foamy” pattern under low magnification, while 34 showed “branch-like” features, both with large interstitial spaces. The rarity of MCP in the malignant group may be because the increased solid components and significant cellular atypia in invasive cancer disrupt the integrity of papillary structures, leading to the disappearance of the microcystic architecture. Additionally, comparison between the two major pathological types of BOETs (serous and mucinous) showed no significant difference in the distribution of the microcystic pattern between the groups.

Our results show a progressive increase in the prevalence of a predominantly solid component, rich blood supply, irregular morphology, and ascites from the benign to the borderline and malignant groups, with statistically significant differences among all three (*P* < 0.001) (**Table 3**). This is consistent with our previous findings on the ultrasound features of ovarian epithelial tumors (37). A key innovation of this study is the combination of the MCP with the “number of positive signs.” The combined diagnostic efficacy of these two was significantly superior to either indicator alone (**Table 5**). While the MCP alone has high specificity (99.0%), its sensitivity is only 53.5% (**Table 5**), leading to potential missed diagnoses in cases with atypical microcystic features. By incorporating other signs like predominantly solid components and rich blood supply, the sensitivity for malignant/borderline differentiation rose to 0.901, and the sensitivity for benign/borderline differentiation also increased substantially, compensating for the limitations of a single feature. This strategy aligns with the “personalized ultrasound diagnosis” concept proposed by the IOTA group (23), but our study is the first to specifically quantify the value of this feature combination for BOETs.

### 4.3 Innovative advantages of the multimodal approach

The most significant advance of this study is the integration of inflammatory markers, tumor markers, and ultrasound features. The AUC for a single serological marker like HE4 in differentiating benign from borderline tumors was only 0.686. However, when combined with PLR/NLR/MLR and ultrasound features, the AUC surged to 0.914. Similarly, for differentiating malignant from borderline tumors, the combined approach achieved an AUC of 0.933, with high sensitivity and specificity (**Table 4** vs. **Table 6**). This breakthrough stems from the complementary nature of multi-dimensional data: ultrasound provides morphological characteristics (like the MCP), inflammatory markers reflect the systemic immune status, and tumor markers indicate the degree of malignant transformation. Chen et al. (38) reported that combining biomarkers with ultrasound features improves the diagnostic performance for distinguishing benign from malignant ovarian tumors, which is consistent with our findings. However, their study did not analyze BOETs as a separate category from malignant tumors. Furthermore, our indicator’s inclusion of inflammatory markers, in addition to traditional tumor markers CA125 and HE4, represents another innovative aspect.

A current clinical dilemma is the frequent misdiagnosis of BOETs as either benign or malignant, leading to either undertreatment or overtreatment (4, 5). The value of our indicators lies in its ability to prevent misclassification as benign, as demonstrated by its 95.0% specificity for benign/borderline differentiation (**Table 6**). Additionally, the inclusion of inflammatory markers can help identify potential malignancy. When CA125 and HE4 are negative in a suspected malignant ovarian epithelial tumor, an elevated NLR (≥2.54) and MLR (≥0.24) can indicate a heightened risk of malignancy (**Table 4**).

### 4.4 Comparison with and advancement of previous research

Asher et al. (39) previously reported on the prognostic role of PLR in ovarian cancer. In our study, the combination of PLR/NLR/MLR with CA125/HE4 achieved AUCs of 0.714 (benign/borderline) and 0.861 (malignant/borderline), significantly outperforming the diagnostic efficacy of the traditional tumor marker CA125 alone (**Table 4**). Huang et al. (28) found that combining CA125 with NLR/PLR was superior to individual markers for identifying BOETs, with high sensitivity and specificity, a finding our study corroborates.

Czekierdowski et al. (13) compared the IOTA Simple Rules Risk (SRR), the ADNEX model, and subjective assessment (SA) with serum CA125, HE4, and the ROMA algorithm for preoperative discrimination among benign tumors, BOTs, and stage I malignant ovarian tumors. They found that diagnostic models based on serum tumor markers CA125 and HE4 and the ROMA (Risk of Ovarian Malignancy Algorithm) had limited value. Among all tests, the ADNEX model had the highest diagnostic accuracy, which was still only 76%. Our combined multimodal approach, which incorporates inflammatory markers and the MCP, improved the sensitivity for identifying BOETs to 0.818 (benign/borderline) and 0.832 (malignant/borderline), with accuracies of 0.885 (benign/borderline) and 0.887 (malignant/borderline) (**Table 6**), thereby addressing the shortcomings of existing models.

### 4.5 Limitations

This study has several limitations. Its retrospective design may introduce selection bias, and generalizability of the study findings needs to be validated in prospective, multicenter studies. Secondly, FIGO staging data were not collected for the borderline ovarian tumor (BOT) and malignant tumor groups in this study. Given that tumor staging is a critical factor influencing preoperative differential diagnosis—clinical characteristics of early-stage ovarian cancer and BOT show greater overlap—this may have introduced bias into the diagnostic efficacy evaluation of some biomarkers. Future studies should incorporate FIGO staging information to further refine the diagnostic efficacy across different tumor stages. Furthermore, the study did not include molecular markers, such as BRAF/KRAS mutations, whose differential expression in serous versus mucinous BOETs could influence biomarker levels (40). Future research could explore the use of artificial intelligence for automated identification of the MCP and integrate it with inflammatory markers to create a real-time predictive tool.

## 5 Conclusion

This study establishes that a multimodal approach combining PLR/NLR/MLR, HE4, CA125, and the ultrasonographic MCP can significantly improve the preoperative differentiation of BOETs. This multimodal approach not only provides a more specific diagnostic tool for these borderline tumors but also elucidates the underlying association between the inflammatory microenvironment and morphological features. Future prospective, multicenter studies are warranted to optimize its cut-off values and validate its clinical utility.

## Data Availability

The original contributions presented in the study are included in the article/supplementary material. Further inquiries can be directed to the corresponding author/s.
